# 4414 Collagen Dermal Replacement Scaffold Mechanobiology in Treatment of Difficult-to-Heal Wounds

**DOI:** 10.1017/cts.2020.58

**Published:** 2020-07-29

**Authors:** David Oleh Sohutskay, Adrian Buganza Tepole, Sherry Voytik-Harbin

**Affiliations:** 1 Purdue University

## Abstract

OBJECTIVES/GOALS: Difficult-to-heal wounds of the skin are a common and costly medical problem. Dermal replacement strategies have emerged as a solution, but a challenge is identification of optimal scaffold parameters. We present a model for assessment of clinical potential of collagen scaffolds for wound healing. METHODS/STUDY POPULATION: In previous animal experiments, we evaluated dermal replacement scaffolds custom-fabricated from fibril-forming collagen oligomer with controlled fibril density (4, 20, 40mg/cm^3^) and spatial gradients in rat excisional wounds. Wound contraction and cellularization were monitored by gross and histological image analysis for comparison with model outcomes. We now parameterize the scaffold parameters for use in the mathematical model of wound healing with nonlinear curve fitting. A preliminary chemo-bio-mechanical finite element model including collagen, cells, and an inflammatory signal was adapted to simulate wound healing results. RESULTS/ANTICIPATED RESULTS: Collagen oligomer microstructure was quantified from scanning electron micrographs. A constitutive law for collagen mechanics was fit to experimental uniaxial tensile tests. We have conducted preliminary three-dimensional finite element model simulations to be validated against experimental wound contraction, recellularization, and collagen remodeling data collected from each experimental group. We show the effects of collagen density and stiffness on wound contraction by altering early wound mechanical properties. We anticipate future work to further improve the model of mechanotransduction, inflammation, and recellularization. DISCUSSION/SIGNIFICANCE OF IMPACT: This work represents the first step towards a computational model of wounds treated with collagen scaffold dermal replacements. In turn, the model will be used to explore cell-scaffold interactions for purposes of prediction and optimization of tissue regeneration outcomes.

